# Cancer diagnosis using label-free SERS-based exosome analysis

**DOI:** 10.7150/thno.92621

**Published:** 2024-02-24

**Authors:** Yajuan Liu, Mei Li, Haisha Liu, Chao Kang, Cheng Wang

**Affiliations:** 1 Key Laboratory of Molecular Target & Clinical Pharmacology, and the NMPA & State Key Laboratory of Respiratory Disease, School of Pharmaceutical Sciences & the Fifth Affiliated Hospital, Guangzhou Medical University, 511436, Guangzhou, China.; 2 School of Chemistry and Chemical Engineering, Guizhou University, 550025, Guiyang, China.; 3 Smurfit Institute of Genetics, Trinity College Dublin, Dublin 2, Ireland.

**Keywords:** Cancer diagnosis, Exosome, Raman Spectroscopy, SERS, label-free SERS

## Abstract

Exosomes, carrying distinctive biomolecules reflective of their parent cell's status and origin, show promise as liquid biopsy biomarkers for cancer diagnosis. However, their clinical translation remains challenging due to their relatively low concentration in body fluids. Surface-Enhanced Raman spectroscopy (SERS) has recently gained significant attention as a label-free and sensitive technique for exosome analysis. This review explores label-free SERS for exosome detection, covering exosome isolation and characterization methods, advancements in SERS substrates, and fingerprint analysis techniques using machine learning. Furthermore, we emphasize the challenges and offer insights into the future prospects of SERS-based exosome analysis to enhance cancer diagnosis.

## 1. Introduction

Extracellular vesicles (EVs), surrounded by a lipid bilayer, are released by both prokaryotes and eukaryotes and can be broadly classified into two main types: microvesicles and exosomes, distinguished by their distinct cellular origins 1. Exosomes originate from endosomes and typically range in diameter from 40 to 160 nm 2. The secretion of exosomes initiates with the formation of multivesicular bodies through plasma membrane invagination 3, 4. Exosome secretion plays a pivotal role in both physiological and pathological processes, influencing the composition of exosomes, including their surface molecules and contents 5. Depending on the specific cell of origin, exosomes carry a wide range of cellular components, such as nucleus acid, lipids, and proteins 5-7.

Exosomes possess a diverse cargo, conveniently accessible in various locations, and packaging essential molecular components, making them exceptionally valuable biomarkers 8. As a superior choice for noninvasive liquid-biopsy in disease diagnosis, prognosis, and monitoring therapy response, exosomes offer promising potential 5, 9-11. Consequently, there is an urgent need for highly sensitive technologies to enhance exosome analysis due to the low concentration of exosomes in biofluid is and to the low recovery efficiency of current isolation techniques, which constitutes a crucial step in exosome analysis 12. In response to this demand, a diverse array of biosensing platforms has emerged, offering promising opportunities to achieve lower limits of detection and significantly enhance the accuracy in exosome characterization 13.

Among the various techniques, surface-enhanced Raman spectroscopy (SERS) stands out as a promising method that significantly amplifies the Raman signal of molecules. This is achieved by attaching the analytes to a plasmonic SERS substrate, resulting in an enhancement factor (EF) of up to 10^11^ 14. The enhancement arises from the interaction of light with nanostructured materials such as gold (Au) and silver (Ag) nanoparticles, which produce a strong surface plasmon resonance effect 14. The unique advantages of SERS, including non-invasiveness, high sensitivity, rapid analysis, resistance to photobleaching and photodegradation, multiplex detection capabilities, and low interference from water, have made it a popular technology in exosome analysis 15.

SERS detection can be broadly classified into two distinct approaches: label-free exosome detection 16 and exosome detection employing SERS-tags 17-20. In SERS-tag analysis, Raman tags, reporters, or dyes are typically affixed to the surface of nanoparticles, which are subsequently bound to exosomes through specific antibodies targeting exosomal biomarkers 21, 22. The sharp and distinct signal emitted by the Raman tag enables precise quantification of exosome concentration, offering exceptional selectivity in detecting exosomes bearing particular biomarkers 13. Labeled-SERS generally exhibits high sensitivity owing to the substantial signal enhancement conferred by labels. This approach provides remarkable specificity, as labeled molecules can precisely target specific exosomal markers. Labeled-SERS finds particular utility in research applications demanding both heightened sensitivity and the precise targeting of specific exosomal components. However, the progress of SERS-tag-based exosome analysis is constrained by the availability of exosome biomarkers and suitable Raman tags 13. Furthermore, labeled-SERS necessitates additional steps for exosome labeling, potentially affecting their biological characteristics. It may also have limitations when applied to high-throughput analysis due to the additional labeling procedures and the associated processing time. Additionally, labeled-SERS can incur extra costs related to the procurement and utilization of labeling agents. The introduction of labels carries the potential risk of altering the biological properties of exosomes, which could be a concern in specific research scenarios.

Conversely, label-free SERS relies on the inherent properties of exosomes, particularly their natural Raman scattering signals 23-26. Label-free SERS provides a unique spectral fingerprint of exosomes without the need for identifying specific exosome biomarkers or employing Raman tags 16. Label-free-SERS simplifies the sample preparation process since it omits the labeling steps typically required 23. This characteristic makes label-free SERS potentially more suitable for high-throughput applications. Moreover, label-free SERS tends to be more cost-effective, as it eliminates the need for external labels. It proves particularly valuable in broader screening contexts or when minimal sample manipulation is desired. Importantly, label-free SERS is less likely to adversely affect the biological integrity of exosomes, rendering it the preferred choice for bioanalytical investigations.

The biochemical characteristics of substances on the surface of exosomes are closely associated with cancer diagnosis 27-29. For instance, the phosphorylation status of proteins may play a crucial role in the early development of cancer cells 28. Numerous research groups have employed different analytical techniques to demonstrate that the degree of protein phosphorylation on the surface of extracellular vesicles isolated from the blood of various cancer patients, including breast cancer, lung cancer, and pancreatic cancer, is significantly higher than that in healthy individuals 27, 28. Furthermore, changes in surface polysaccharides 29 and lipid molecules (such as phospholipids, cholesterol, sphingolipids, and ceramides) 30 can also be used to detect pancreatic, lung, liver, and colon-related cancers. These studies indicate that cancer occurrence is closely linked to the biochemical changes of many substances on the surface of exosomes, rather than relying solely on a single protein marker. Label-free SERS enables the characterization of exosomes by capturing their unique fingerprint, which corresponds to the specific biochemical compounds present on their surface. Based on this advantage, label-free SERS has demonstrated promising applications in diagnosing various types of cancers, including lung cancer 31-33 breast cancer 34-37, melanoma 38, oral and oropharyngeal squamous cell carcinoma 39, head and neck cancer 40, urogenital cancer 41, hepatocellular carcinoma 42, nasopharyngeal cancer 43 and gastric cancer 44. Moreover, this technique allows for simultaneous diagnosis of multiple cancers 28, 45 and can accurately identify cancer in patients at different stages 46.

This review primarily explores exosome detection using label-free SERS. We began by discussing exosome isolation and characterization methods from label-free SERS-based exosome research, as the concentration and size of exosomes are crucial factors in SERS analysis. Next, we focused on the advancements in SERS substrates, as their selection and improvement play a pivotal role in SERS analysis. While label-free SERS technology offers the potential for simultaneous analysis of various diseases, there is a risk of signal overlap among different biomolecules, leading to the loss of some diagnostic signals. Consequently, we also summarized data analysis methods, particularly fingerprint analysis techniques, which include linear/non-linear machine learning methods commonly utilized in label-free SERS analysis. We concentrate on research from the past 5 years, highlighting challenges and providing insights into the future potential of SERS-based exosome analysis for improving cancer diagnosis.

## 2. Exosome isolation and characterization

**Figure 1:**
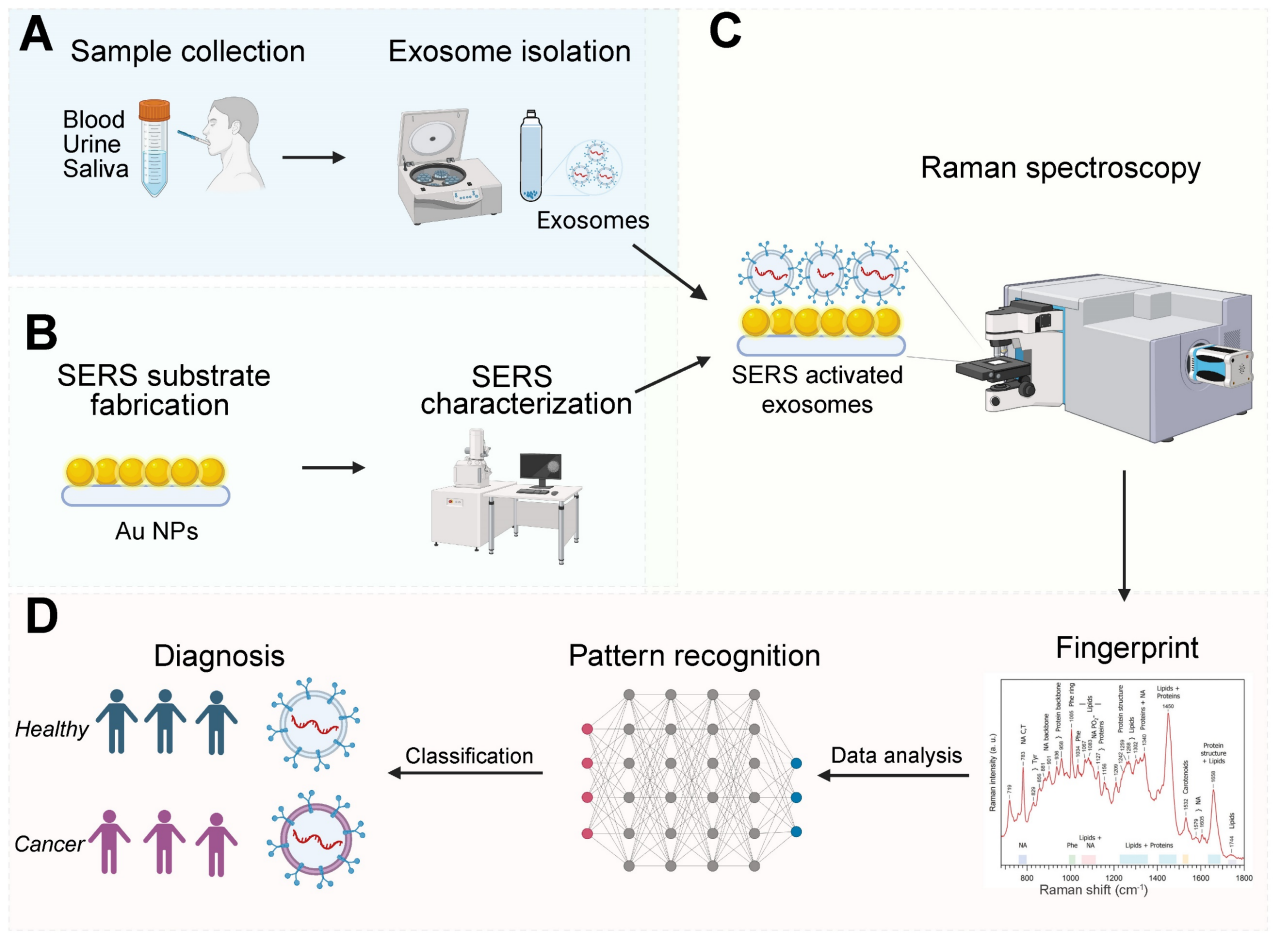
Illustration of exosome analysis for cancer diagnosis using label-free SERS. (A) Sample collection, exosome isolation. (B) Fabrication and characterization of SERS substrate. (C) Spectroscopic data collection of exosomes via SERS. (D) Exosome classification and cancer diagnosis through SERS patterns. The Figure 1D fingerprint image was adapted with permission from reference 79, copyright 2012 Wiley.

For effective SERS analysis of exosomes, it's essential to isolate exosomes specifically from an extensive range of body fluids, including blood/plasma/serum 28, 33, 36, 40-42, 44-52, urine 53 and saliva 39. Depending on the research purpose and experimental conditions, exosomes may also be isolated from other sources such as fetal bovine serum 54 and bronchoalveolar lavage fluid 31. It should be noted that cells 23, 32-35, 37, 38, 43, 46, 49, 55-60 are also commonly used for discriminative analysis of cancerous and non-cancerous exosomes based on SERS (Figure 1). Significant advancements have been achieved in the field of exosome isolation techniques, yielding promising insights into the understanding of exosomes 61, 62. However, it has become evident that the rapid and efficient isolation of exosomes remains a challenge, primarily due to the intricate nature of biological samples, the potential interference from other extracellular vesicles sharing similar physicochemical and biochemical properties, and the inherent heterogeneity of exosomes themselves. A variety of methods (Table 1) have been employed to successfully extract exosomes from a wide range of sample matrices, showcasing their effectiveness.

Ultracentrifugation is widely regarded as the gold standard for isolating exosomes due to its ability to generate exceptionally high centrifugal forces 61. The centrifugal force employed typically falls within the range of approximately 100,000 to 120,000 times. It is a user-friendly approach, as it does not demand extensive technical knowledge. It offers long-term affordability, as only one ultracentrifuge machine is needed for extended use. Additionally, it is a moderately time-consuming method that typically does not require complex sample pretreatments. Consequently, ultracentrifugation (UC)-based techniques have gained significant popularity in SERS analysis exploring exosomes 28, 32-34, 38-40, 42, 48, 51, 56, 57, 59, 60, 63. Size exclusion chromatography (SEC) 31-33, 36, 40, 45-47, 55 and ultrafiltration 37, 38, 41, 58 are popular size-based exosome isolation techniques. Some commercial assay kits which are based immunity capture are also commonly used in exosome isolation techniques 33, 35, 49, 54. It is worth mentioning that a magnetic SERS platform has been developed to facilitate the combined process of exosome isolation and Raman signal enhancement. It is noteworthy that a magnetic SERS platform has been developed to streamline the concurrent processes of exosome isolation and Raman signal enhancement 23, 50. This innovative system integrates a microfluidic Raman biochip for the isolation and detection of serum exosomes on a single chip, with Raman beads being employed to differentiate between individuals with good health and those diagnosed with cancer, all within the span of 1 h. This device is capable of efficiently handling and detecting low-volume samples within a short timeframe, with a median isolation efficiency.

To assess the quality of isolated exosomes, numerous techniques have been developed for characterizing their protein content, size distribution, morphology, concentration, and biochemical composition. Nanoparticle tracking analysis (NTA) are commonly used to count and size exosome, which are viewed by the incident illumination coming from laser light 32, 36-40, 45-48, 50-52, 55, 57-59, 63. Transmission electron microscopy (TEM) is used as a compensation to confirm the detection of NTA as it is relatively time-consuming and not fitted for a large amount of exosomes although it can be used to check the quality of preparations in a visualized way 28, 31, 33, 36-38, 40-43, 45-47, 50, 51, 53, 54, 56-60. Western blotting is an important procedure for exosome characterization to confirm some specific proteins in exosomes 28, 32, 33, 36, 41, 43, 45, 46, 48, 50, 53, 54, 56-60. As well as those commonly used techniques, there are some more techniques used for exosome characterization depending on the research purpose before SERS analysis, such as dynamic light scattering 32, 34-36, 38, 47, 60, zeta potential analyzer 32, 38, fluorescence 23, flow cytometry 43, 52, 58, atomic force microscope 48, single particle interferometric reflectance imaging sensing 40, tunable resistive pulse sensing 54 and protein assay kit 60.

## 3. SERS Substrate

### 3.1 Evaluate the SERS effect

Regarding the SERS effect, it is of utmost importance to characterize the NPs, which involves assessing their size, shape, and distribution. Various microscopy techniques, including scanning electron microscopy 23, 28, 31, 32, 34-36, 40, 45, 46, 49, 53-57, 63, transmission electron microscope 23, 33, 38, 41, 49, 50, atomic force microscopy 34, 55, 63. have been employed for this purpose. These microscopies not only aid in estimating the uniformity and average number of NPs, but also provide valuable insights into their overall structure. 45. Furthermore, multiple spectroscopic methods have been widely used to characterize and confirm the formation of NPs. Notable among these are UV-visible spectrophotometry 28, 34, 38, 43, 53, 60, X-ray spectroscopy 23, 35, 53, 56, dynamic light scattering 38, electrochemical impedance spectroscopy 35, Fourier transform infrared spectroscopy 56. For instance, UV-Vis spectrophotometry allows monitoring the characteristic surface plasmon resonance (SPR) band of NPs, confirming their formation 34. In some cases, researchers have employed multiple techniques to ensure thorough verification 35. Combining different characterization methods enhances the reliability and accuracy of the results, providing a comprehensive understanding of the NPs under investigation.

In the realm of SERS substrate design, researchers have harnessed the power of the FDTD method, a robust numerical tool for computational electrodynamics modeling 23, 28, 38, 49, 53-55, 57. By employing FDTD, they have gain profound insights into the intricate interplay between electromagnetic (EM) fields and the localized surface plasmon resonances (LSPRs) of the substrate 67. This advanced simulation technique has enabled both qualitative and quantitative analyses of SERS phenomena, fostering a more comprehensive understanding of the underlying physics 68. Moreover, FDTD has emerged as a highly valuable tool for optimizing the design and fabrication of SERS substrates, facilitating precise molecular analysis for diverse applications.

To assess the SERS effect, EF is one of the most important metric to gauge the "magnitude" of the enhancement in SERS 45, 55. Indeed, the substantial variation in the reported EFs can be attributed to a combination of factors, notably the wide variability in the EF's definition and the diverse methods utilized for its estimation in practice 28, 34, 35, 43, 49, 57, 67. Such discrepancies in the definitions and equations of EFs emerge from varying perspectives, including the single molecule viewpoint, SERS substrate viewpoint, and analytical chemistry viewpoint 69. Besides, EF calculation from different research are based on different materials such as methylene blue (MB) 28, rhodamine 6G (R6G) 34, 45, 4-mercaptobenzoic acid (4-MBA) 49, crystal violet 35, exosome 38, 42, 50, 55. It should be noted that a vague EF emerges, wherein essential normalization concerning non-SERS conditions is occasionally disregarded, a factor of significant importance.

### 3.2 Basic SERS substrate

SERS substrate based on various materials which significantly enhances the Raman signal is one of the keys to guarantee the sensitivity of the label-free exosome analysis (Figure 1). Several criteria are important when selecting a SERS substrate for exosome analysis. Firstly, it should exhibit high sensitivity, given the typically low concentration of exosomes. Secondly, it should minimize any adverse effects on the biological activity of exosomes. Gold (Au) is the preferred material for exosome analysis due to its strong enhancement effect and minimal impact on the biological activity of exosomes 37, 40, 58, 60, 70. Silver (Ag) is occasionally used as an alternative because it can provide even stronger absorption effects 39, 44, 59. However, the use of Ag may potentially diminish the biological activity of exosomes. Based on those materials, the simple way to construct SERS substrate is to drop exosome and NPs evenly on the silicon 37/CaF_2_ 39 substrate. The NPs can be either self-made 37 or commercially available 40. The ratio between NPs and exosomes are based on their concentrations. The NPs cannot always be guaranteed to successfully assembled which results in low and subsequent uniformity of SERS substrate and low experimental repeatability. The SERS signal has been improved in various ways.

### 3.3 Size and shape of NPs

Multiple studies have demonstrated a correlation between the SERS signal and the size and shape of NPs 71-73. Consequently, one viable approach to enhancing the SERS signal is by exerting control over the size and shape of NPs. In the context of exosome analysis, spherical NPs are commonly employed (either self-prepared or purchased from different suppliers). The size of these NPs varies across different studies, typically ranging from 20-200nm (refer to Table 2). It is worth noting that the SERS signal could potentially be influenced by both the size of exosomes and the NPs themselves, although our understanding of this relationship remains incomplete.

**Figure 2:**
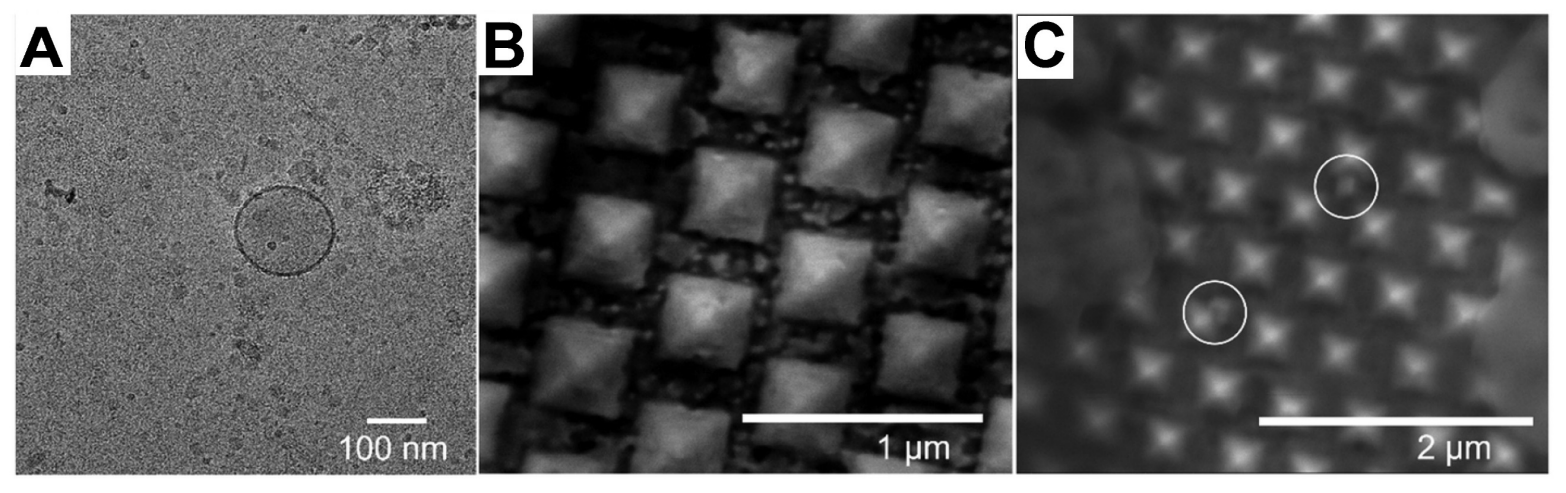
(A) TEM image of EVs (B) SEM image of the SERS Au nanopyramid substrate. (C) SEM image of the SERS Au nanopyramid substrate after introduction of EV. Adapted with permission from reference 31, copyright 2023 Royal Society of Chemistry.

SERS analysis has been performed on exosomes using various shapes of nanoparticles. For instance, Au nano stars with a tip-to-tip size of approximately 71.0 nm were synthesized and utilized for exosome analysis in breast cancer diagnosis and postoperative assessment, taking advantage of their strong plasmonic properties for SERS enhancement 36. Additionally, inverted Au nanopyramids with sidewalls at specific angles were created, establishing a correlation between the sizes of "hotspots" and the sizes of small EVs (∼80-150 nm), which can be seen the TEM image of EV in Figure 2A 31. The SEM images in depict the SERS substrate before (Figure 2B) and after (Figure 2C) the introduction of EV, which illustrate that the vesicles are situated between individual nanopyramids. This design enabled the substrate to capture SERS signals from individual vesicles one at a time. Furthermore, a hybrid SERS substrate consisting of graphene covered by Au nanopyramids was developed for the identification of exosomes originating from different sources 54. In this particular study, a patterning method utilizing a layer of self-assembled polystyrene balls was employed to fabricate a quasi-periodic structure of gold nanopyramids with base dimensions of approximately 200 * 200 nm². Finite-difference time-domain method (FDTD) simulations demonstrated that the nanopyramids' sides contained "hotspots" with significantly enhanced electromagnetic fields.

### 3.4 Core-shell structure

Core-shell nanostructures comprising multiple components have proven to be exceptionally effective as SERS substrates for sensitive exosome analysis, as they can maintain a robust enhancement effect while exerting minimal influence on the biological activity of exosomes. Among these strategies, the Au@Ag core-shell nanostructure is commonly employed to enhance detection sensitivity 74. For instance, the Au@Ag substrate exhibits remarkable sensitivity when utilized on a 2D hydrophobic substrate, with an EF of 4.57*10^7^ 43. In addition to generating higher SERS intensities through the plasmonic properties of the core-shell structure, the silver layer also serves the purpose of eliminating interference from the coating agent molecules in the SERS spectra 38. In this specific study, AuNPs were functionalized with 4-dimethylaminopyridine (DMAP) molecules to facilitate the attachment of EVs. However, DMAP itself exhibits a significant SERS signal, which can potentially interfere with the SERS signal from the EVs. To mitigate this issue, an additional silver layer was added to form Au@Ag core-shell NPs. This not only eliminates the interference caused by the DMAP molecules in the SERS analysis but also enhances the SERS signal due to the plasmonic properties of the core-shell structure. To be noted, the drying process is not required prior to SERS analysis in this case. Instead, the solution containing SERS-enhanced exosomes can be directly measured using a water dipping objective. In addition to the Au@Ag core-shell structure mentioned earlier, magnetic core-shell structures are also commonly employed in exosome analysis. These structures integrate cancer exosome isolation and Raman signal enhancement into a single system. Typically, the magnetic core-shell structure is used in conjunction with a Raman reporter and antibodies 50. At the same time, the magnetic core-shell structures are also used in label-free strategy. For instance, superparamagnetic Fe_3_O_4_ NPs serve as cores for separation, while the Au layers enable label-free SERS detection 23. This approach effectively distinguishes exosomes from different cell sources for cancer diagnosis.

### 3.5 Tailored substrate materials

**Figure 3:**
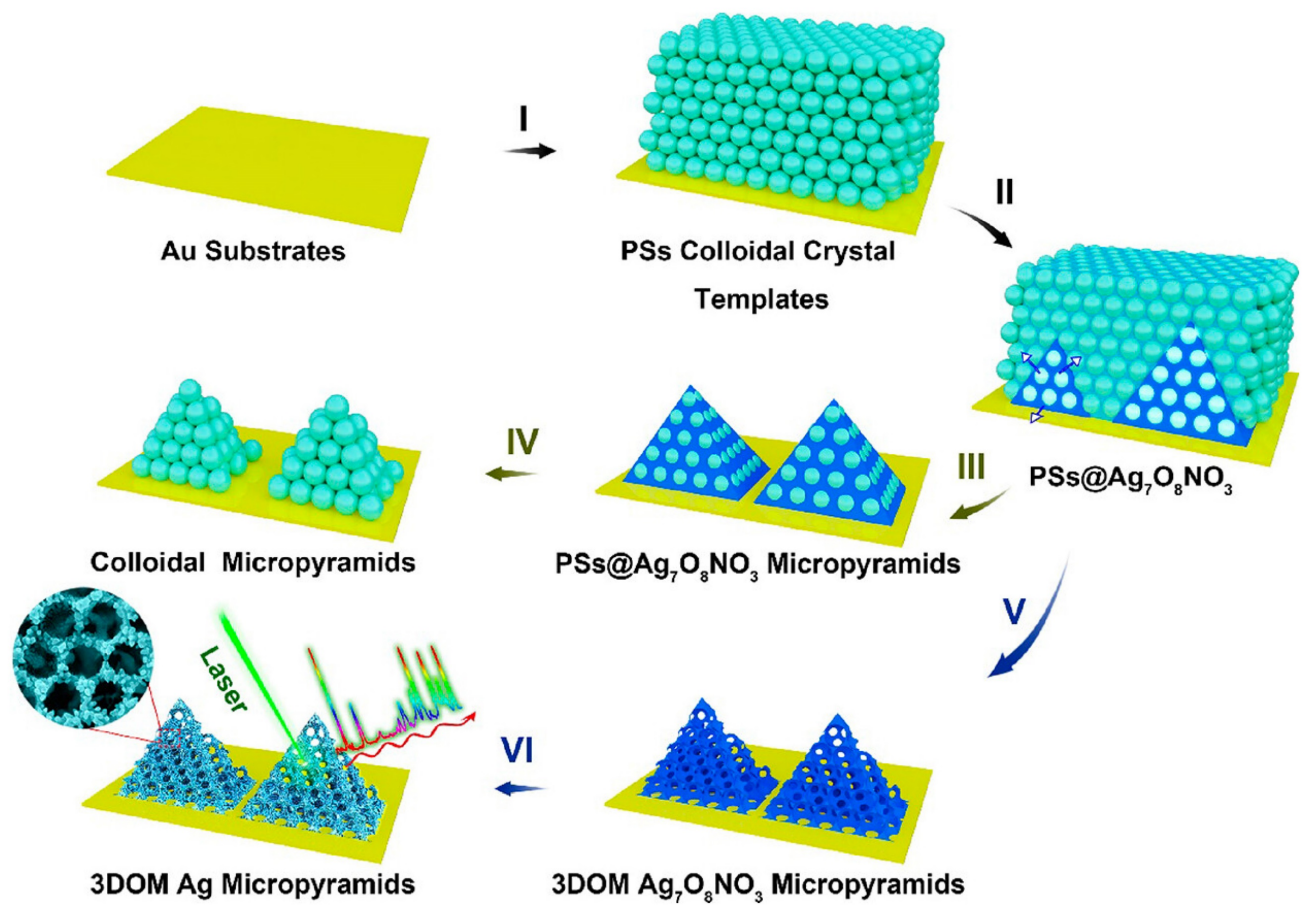
Schematic depicting the electrochemical cementing technique for the synthesis of 3DOM Ag_7_O_8_NO_3_ micropyramids, followed by their shape-preserving conversion into 3DOM Ag micropyramids. Adapted with permission from reference 40, copyright 2021 Royal Society of Chemistry.

As mentioned before, silicon 37/CaF_2_ 39 substrate is a common base used to contain the NPs in SERS analysis. In the same time, to improve uniformity and SERS signal, researchers have developed various bases, such as quartz microfiber matrix 40, hydrophobic substrate 43, 54, bacterial nanocellulose 34, 3D plasmonic ITO glass 49, platinum-black (Pt-black) template 35, and porous substrate 28. Porous substrate is one of the strategies to improve the SERS signal. An engineered 3D Au-coated TiO_2_ macroporous inverse opal (MIO) structure was developed, exhibiting an impressive EF of 1.2*10^5^ 28. The 3D Au-coated TiO_2_ MIO structure shows advantages of detecting large exosomes, primarily through their interconnected nanoscale pore networks. Additionally, the TiO_2_ MIO structures demonstrate a remarkable “slow light effect” leading to a substantial enhancement of Raman signals from exosomes. Another example of the porous substrate includes 3D ordered macroporous (3DOM) Ag_7_O_8_NO_3_ micropyramids, which were formed by AgNPs through a chemical reduction process 57 (See Figure 3). The interconnected macropores within these micropyramids play a crucial role in facilitating the transportation and enrichment of analyte molecules. Additionally, the dense distribution of AgNPs on the micropyramid skeletons results in the generation of strong electromagnetic fields. Consequently, the 3DOM Ag micropyramids serve as highly efficient single-particle SERS sensing substrates, showcasing remarkable SERS sensitivity and signal reproducibility. Moreover, employing a photolithography process on Si substrates, a precise hexagonal array of circular holes was fabricated 52. The primary objective of this process was to construct microstructured arrays that could effectively encapsulate AuNPs. Significantly, these microstructured Au arrays have demonstrated their utility in the stratification of multiple myeloma patients through exosome profiling.

**Figure 4:**
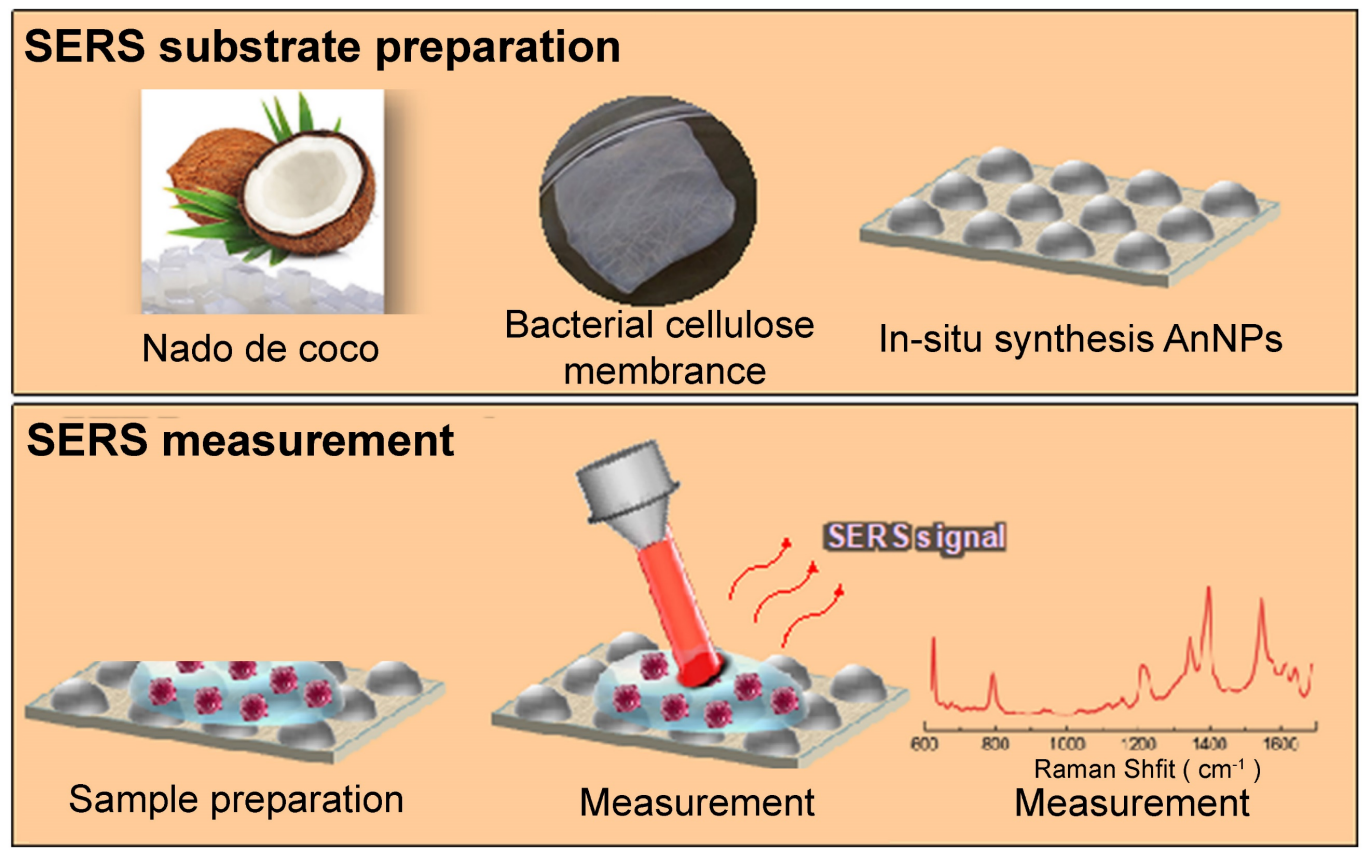
Schematic illustration of the SERS substrate fabrication using BC. Upper section: Production of BC membrane using commercially available nata de coco cubes, accompanied by the in-situ synthesis of AgNPs within the BC matrix for generating SERS substrates. Lower section: Preparation of the SERS assay, measurement process, and resulting spectral analysis. Adapted with permission from reference 34, copyright 2019 American Chemical Society.

Comparable to a porous structure, a quartz microfiber matrix containing AuNPs was affixed onto a borosilicate glass substrate for the purpose of conducting SERS measurements 40. Analyzing the SEM images revealed the even distribution of EVs within this matrix, with a specific concentration in close proximity to the plasmonic gold, thus enabling exposure to intense electromagnetic fields resulting from the interaction between the excitation laser and the localized surface. In addition, bacterial nanocellulose produced from commercial nata de coco, combined with in situ synthesized AgNPs, has been tested as a cost-effective and environmentally friendly SERS substrate. In Figure 4, a schematic diagram depicts the process of creating the SERS substrate through the utilization of bacterial cellulose (BC). This approach has yielded impressive EF ranging from 10^4^ to 10^5^ 34. Besides, a custom Pt-black SERS template has been developed and compared to the commercially available substrate (SERS-Au) 35. The Pt-black SERS template exhibits stable, consistent, and low background spectra, resulting in the high reproducibility essential for a reliable diagnostic template, even though it achieves a lower EF compared with SERS-Au. In addition to Pt-black, ITO glass has also been utilized to create 3D plasmonic AuNPs nanomembranes as substrates, which have proven to be successful in cancer diagnosis and dynamic monitoring of drug therapeutic processes 49.

### 3.6 Linking molecules

One strategy to enhance the uniformity of the SERS signal is by employing a linking molecule to connect the NPs and exosomes. A highly effective and straightforward approach to achieve a uniform and reproducible SERS substrate is by using the 3-aminopropyl triethoxy silane (APTES)-functionalized SERS substrate 32, 33, 45, 46, 75. This is because films formed by silane coupling agents allow for the deposition of coatings with adjustable thickness, roughness, and well-defined chemistry.

Antibodies are widely utilized to enhance the binding capability between NPs and exosomes. In many cases, a common strategy involves labeling with specific antibodies and a particular Raman reporter. Additionally, antibodies are also employed for label-free SERS analysis. For instance, a study utilized epidermal growth factor receptor (EGFR) antibody-coated glass to connect AuNPs and exosomes 33. In this research, APTES was also utilized as a linking molecule. Another example of using antibodies as linking molecules in label-free exosome analysis involves SERS analysis based on AuNPs through polyethylene glycol and bio-receptors 42. Besides, cysteamine also serves as a valuable biofunctionalization linker. It features a terminal thiol group at one end, facilitating binding to the gold substrate. At the opposite end, there is a free amine that effectively enhances the surface with a positive charge. This charge modification enables the non-specific capture of inherently anionic EVs 40. In addition, DMAP can also serve as a linking molecule between NPs and exosomes. AuNPs have cationic surface charges attributed to the DMAP coating, allowing them to adsorb onto the anionic EV surface. This association is primarily charge-based, although there is a possibility that DMAP molecules interact with cysteine-rich proteins found on the EV surface 38.

### 3.7 Microfluidic device

**Figure 5:**
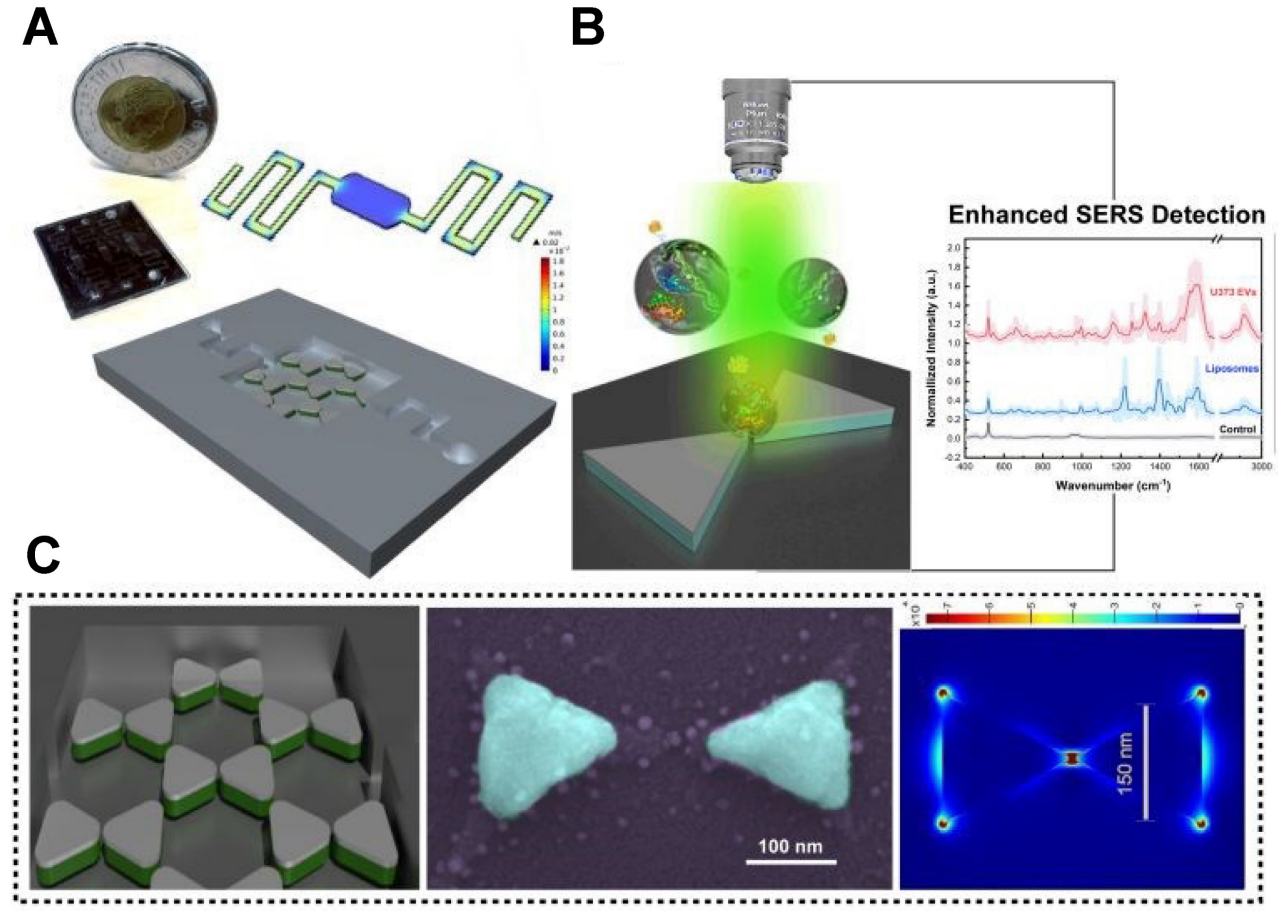
Utilizing a spatially suspended nanobowtie surface within a microfluidic device for enhanced SERS-based detection of EVs. (A) Design and prototype of the microfluidic setup. (B) EV detection using SERS methodology. (C) Nanobowtie design and corresponding artificially colored SEM image of the produced nanobowtie structure (Cyan). The final section showcases FDTD simulations displaying the distribution of maximum electromagnetic field intensity on the surface of a nanobowtie within a honeycomb array. Adapted with permission from reference 55, copyright 2021 Royal Society of Chemistry.

Microfluidic technology can achieve high-throughput separation and enrichment of exosomes, while addressing some core issues in SERS detection 76. For instance, microfluidic SERS technology can precisely control the aggregation time of SERS NPs and exosomes by adjusting flow rates and designing channel structures, thereby improving their mixing efficiency and addressing the issue of poor reproducibility in SERS analysis 22. Moreover, it enables high-throughput sample preparation to achieve a more stable and reliable analytical detection 77. Most of the current microfluidic SERS technology is primarily based on immune probe capture and detection of specific tumor markers in exosomes, unable to obtain a complete fingerprint spectrum 76. In the context of label-free analysis, Jalali et al. introduced a pioneering method for exosome fingerprinting by innovatively combining a plasmonic nanostructure with a microfluidic sample delivery system 55. They strategically positioned nanobowties at the bottom of the fluidic chamber, taking advantage of their strong electromagnetic field (EF) enhancement capabilities, which facilitated the uniform distribution of exosomes next to the plasmonic surface (Figure 5A-B). The final panel in Figure 5C illustrates FDTD simulation depicting the distribution of the maximum EF on the surface of a nanobowtie arranged within a honeycomb array. The study successfully demonstrated the synergistic combination of nanosurface microfluidics and the powerful plasmonic effects of suspended nanobowties, leading to the creation of a highly sensitive label-free SERS detection device for exosomes.

## 4. Data analysis

### 4.1 Raman peak assignment

The exosome's surface comprises a complex biochemical composition of lipids, nucleic acids, and proteins. The intricacy of this composition is reflected in the complex Raman peaks observed during analysis. Assigning these peaks accurately is crucial for understanding the chemical composition of the sample. These peak assignments are generally based on various references, including Raman analysis of exosomes 60, 78, 79, SERS-based exosome analysis 32, 38, 80, SERS analysis of body fluid 81-88, and some Raman analysis of body fluid/tissue 89-103, and review paper 104-106. Besides, it is important to note that label-free SERS peak assignments are related to the spontaneous Raman peaks of exosomes. However, several factors such as the type of exosomes, laser wavelength, SERS substrate, solvent, etc., can influence the actual peak assignment. Consequently, Raman profiles and peak assignments may vary across different research studies 38, 39, 54, 55, 58. Table 3 provides a list of common peak assignments related to important biomolecules used in exosome characterization. Given that the primary composition of the exosome surface is the lipid bilayer, many of the assigned peaks correspond to lipids and related subpopulations, such as fatty acids, cholesterol, and phospholipids. Additionally, proteins, nucleic acids, and related compounds are frequently utilized for exosome characterization in various research studies. Furthermore, glycoproteins 39, 58 and phosphoproteins 28 are used as biomarkers due to their significance in cancer development. Consequently, identifying chemicals like saccharides and phosphate groups becomes crucial. Apart from specific Raman peak assignments, the entire spectrum of certain biomarkers is also employed in exosome characterization. For example, Shin et al. utilized the fingerprint of common protein biomarkers CD9 and CD81, which are expressed on the surfaces of all exosomes, along with cancer-specific protein biomarkers like EGFR expressed in non-small cell lung cancer exosomes 32. Comparing the spectral similarities between the protein biomarkers and PCA-decomposed spectra of exosomes allowed for the discrimination between normal and cancerous exosomes. It is crucial to relate to biomolecules/biomarker to the corresponding Raman peaks because it provides essential information about the exosome's molecular makeup and can lead to a better understanding of their biological roles, disease biomarkers, and potential therapeutic applications.

### 4.2 Univariate analysis

Univariate analysis in SERS analysis refers to the interpretation of individual spectral peak independently, without considering the interactions or correlations between different peaks. The first step of the univariate analysis is to assign Raman peaks to specific molecular vibrations and select the characteristic peak of exosome. Once the characteristic peak is selected, statistical methods are generally applied to discriminate the normal and cancerous exosome sample. For instance, research has revealed that the SERS intensity of exosomes at 1087 cm^-1^, attributed to the P-O bond within phosphoproteins, can serve as a valuable marker for tumor liquid biopsies 28. A *t*-test analysis was performed, demonstrating a significant difference in Raman intensity between plasma exosomes from patients with prostate cancer and those without the condition. In addition to utilizing a single peak, univariate analysis can also involve examining the ratio between two peaks. For instance, the ratio R=*I*_2880_/*I*_2930_ is used to assess the packing density of the lipid membrane in the CH region 55. This peak intensity ratio serves as an indicator of the lateral packing density of the acyl chain in the CH region and, consequently, reflects the level of conformational arrangement and interchain coupling within the lipid structure. Univariate analysis plays a crucial role as a starting point in SERS studies, and it can be utilized for discrimination analysis in certain cases. However, to achieve a more comprehensive understanding, researchers often turn to multivariate analysis techniques. These methods are employed to explore the interdependencies and correlations among multiple spectral features within complex SERS datasets, particularly due to the intricate SERS fingerprint of exosomes. By utilizing multivariate analysis, researchers can uncover more intricate patterns and extract valuable insights from the data.

### 4.3 Linear machine learning methods

**Figure 6:**
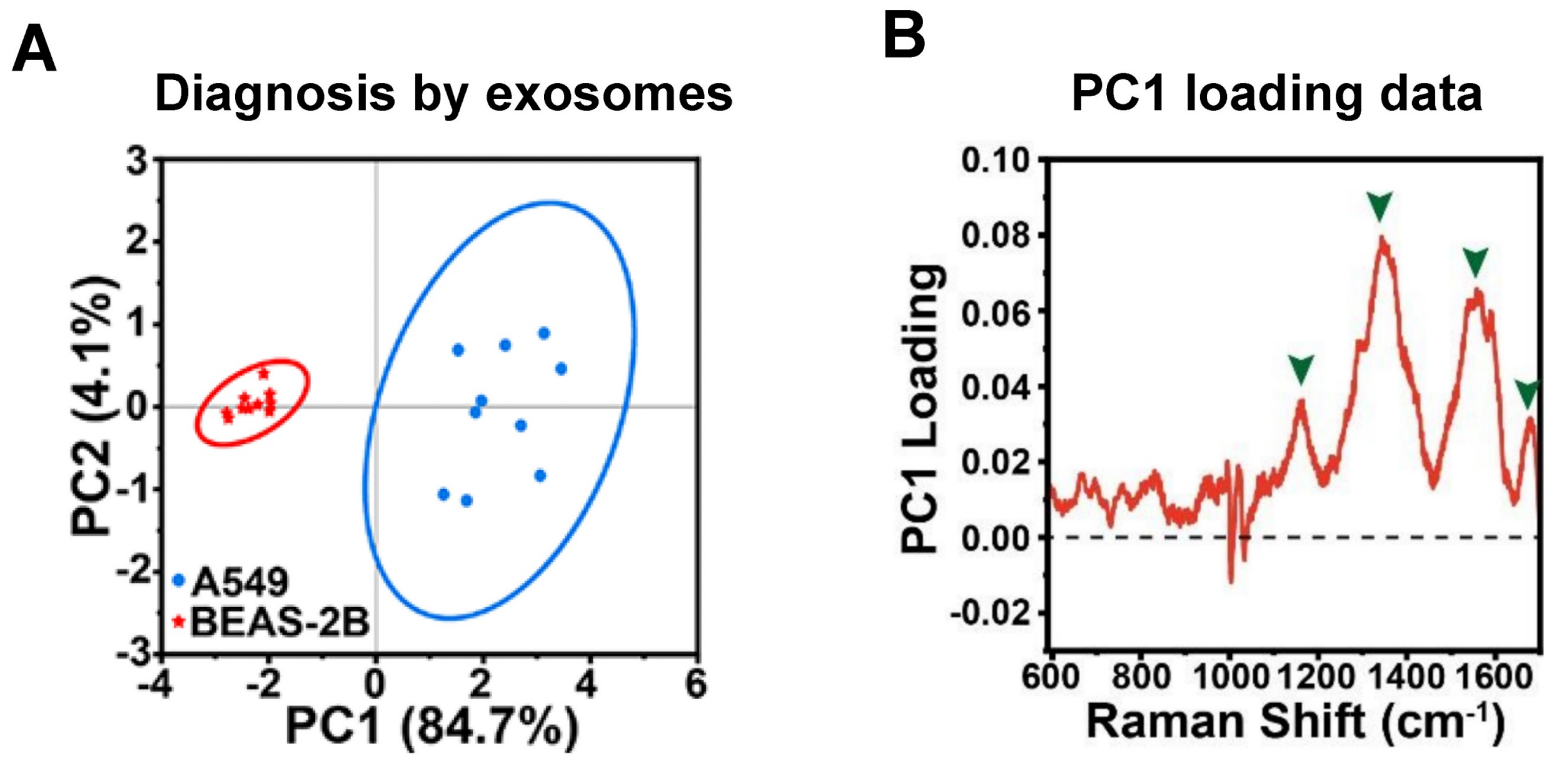
(A) PCA score depicting the SERS spectra of exosomes derived from two distinct cell lines. (B) PC1 loading plot of PCA score plot from Figure 6A. The enclosed circles correspond to the 95% confidence ellipses. Adapted with permission from reference 57, copyright 2023 American Chemical Society.

Principal component analysis (PCA) is a widely employed technique in label-free SERS-based exosome analysis, aimed at reducing data dimensionality while retaining crucial information 23, 32, 34, 50, 54, 57. By transforming the data into a new set of orthogonal loadings, PCA extracts the essential features from the original spectra. The sample projections on these Principal components (PCs) represent a score matrix, revealing distinct clustering patterns among the samples. For instance, in this study, PCA was performed on SERS analysis to classify A549 cell-derived and BEAS-2B cell-derived exosomes 57. The loading plot (Figure 6A) indicated that four main SERS peaks (i.e., 1159, 1343, 1557, and 1678 cm^-1^) with high loading values contributed significantly to PC1, and these peaks were associated with DNA and proteins). This suggests that the unique proteins and DNA found in the A549 cell-derived exosomes played a crucial role in distinguishing between the two groups. Consequently, the PCA score plot (Figure 6B) demonstrated that the two types of exosomes exhibited significant differences and were clustered into separate groups, validating the effectiveness of PCA in accurately classifying the samples based on their molecular content. PCA is a powerful tool in SERS analysis, offering various benefits such as simplifying complex spectral datasets, enabling visualization, improving signal-to-noise ratio, and assisting in feature extraction and pattern recognition tasks. However, it is worth noting that PCA is a relatively simple linear unsupervised method. As a result, it is often utilized primarily for feature extraction and visualization. To achieve more precise classification results, researchers commonly incorporate more sophisticated methods independently or after applying PCA to the data and extracting relevant features. These advanced techniques may include support vector machines 31, 42, linear discriminant analysis (LDA) 39, 40, 42, 49, partial least squares discriminant analysis (PLS-DA) 38, 49. By leveraging these supervised pattern recognition methods, researchers can capitalize on the simplified and meaningful representations obtained from PCA, allowing for more accurate and comprehensive analyses, especially when PCA is used as an initial step. This integrated approach helps to obtain a more robust and insightful analysis of the SERS data, leading to a deeper understanding of the underlying patterns and structures within the dataset.

### 4.4 Deep learning methods

**Figure 7:**
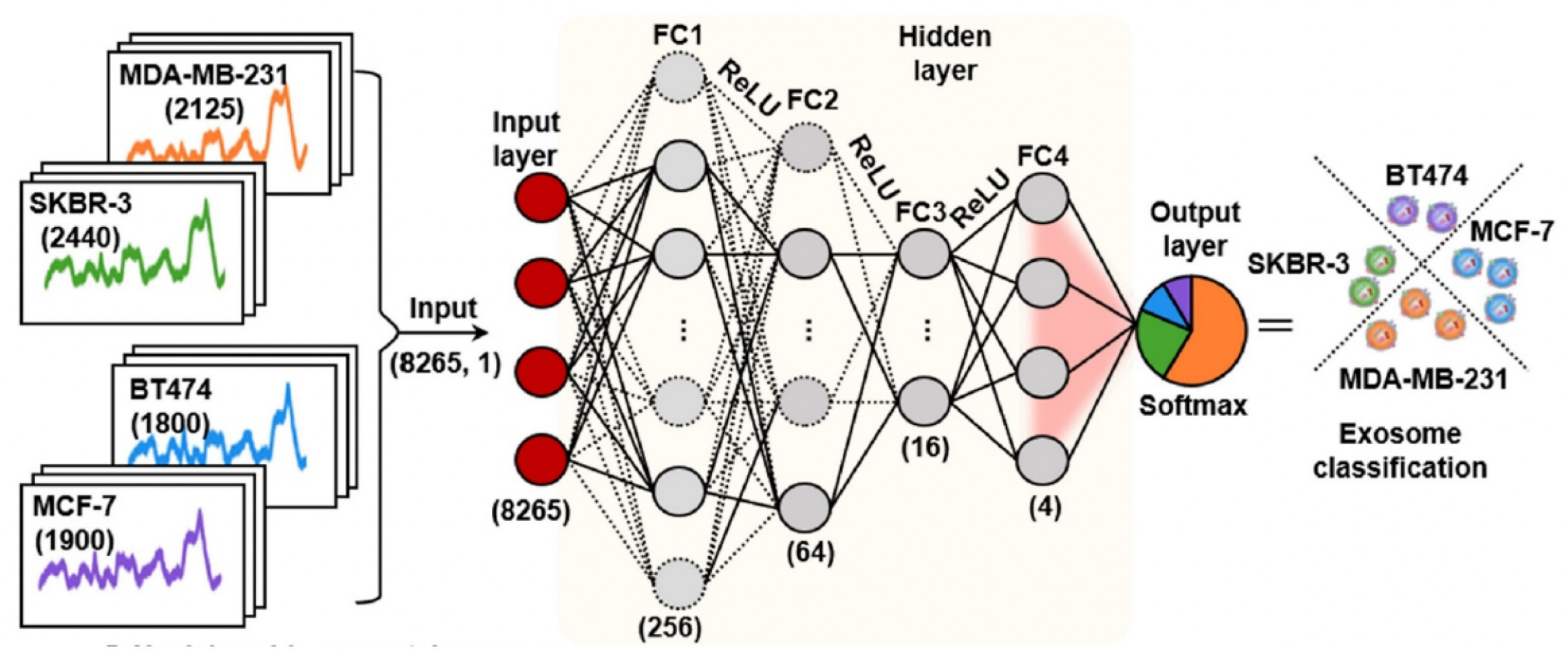
The CNN model's architecture comprises an input layer, four fully connected hidden layers, and an output layer. This arrangement produces a conclusive output which represent the predictive probabilities corresponding to each category of cell-derived exosomes. Adapted with permission from reference 36, copyright 2022 American Chemical Society.

Deep learning use artificial neural networks with multiple layers to learn and represent complex patterns in data, which shown significant promise in SERS analysis, as it can handle large and complex spectral datasets, extract meaningful features, and perform accurate classification or regression tasks 33, 35-37, 41, 43, 45-48. Convolutional neural network (CNN) is a specialized type of artificial neural network designed which is one of the most popular deep learning methods used in the SERS analysis. One of the CNN architecture was shown in Figure 7 36. In this study, CNN successfully predicted whether exosome samples originated from breast cancer patients and accurately assessed different subtypes of breast cancer through the SERS signals of exosomes derived from normal and breast cancer cells, achieving a prediction accuracy of 100%. The increasing number of exosome research samples has enhanced the potential of deep learning for clinical analysis 15. In their study using 520 test samples, Shin et al. not only detected early cancer but also accurately identified the presence of multiple cancer types based on CNN, including lung, breast, colon, liver, pancreas, and stomach, with an impressive area under the curve value of 0.970 based on the same database. The evidence provided by multiple research studies consistently demonstrates that deep learning consistently outperforms linear analysis methods like PCA, delivering superior and more precise results in various applications 41, 43, 46, 48. Label-free SERS-based exosome analysis with deep learning exhibits tremendous potential, offering enhanced accuracy, automation, and scalability for processing vast and diverse datasets 33, 35-37, 41, 43-48. For example, Wu et al. demonstrated higher accuracy using Backpropagation BP neural network models (92.4%) compared to PCA-LDA (64.7%) in their SERS-based analysis for evaluating nasopharyngeal cancer radioresistance 43. Despite challenges in widespread deep learning adoption, including resource requirements and training data, continuous advancements in techniques and growing SERS-based exosome databases promise significant progress in this field. We have summarized the pros and cons of various data analysis methods in Table 4.

## 5. Conclusion and outlook

In this comprehensive review, our primary emphasis is placed on label-free SERS-based exosome detection. Our aim is to provide valuable insights into the current endeavors aimed at addressing pivotal challenges and to delve into innovative avenues for SERS-based exosome analysis, particularly within the realm of cancer diagnosis and beyond. The exploration begins with a thorough examination of exosome isolation and characterization methods, emphasizing their importance in SERS analysis due to the influence of exosome concentration and size. The significant and impactful advancements in SERS-based exosome research can only be realized through the development of highly efficient exosome isolation techniques. A meticulous selection of isolation methods tailored to specific biological samples and cargo types being screened will not only enhance the quality of isolated exosomes but also ensure the validity of the SERS results. The integration of microfluidic devices with label-free SERS analysis automation represents a compelling approach to streamline the isolation and analysis process. This integration has the potential to reduce sample volume requirements and significantly improve the overall efficiency and reliability of label-free SERS-based exosome research. Furthermore, the development of magnet SERS substrates presents an intriguing technique that combines exosome isolation and detection. This novel approach holds promise in high-throughput SERS analysis, opening exciting possibilities for advancing exosome research.

However, despite its potential, SERS-based label-free exosome detection faces several significant challenges that must be addressed for it to fully serve clinical and research applications. Exosomes exhibit a remarkable degree of heterogeneity concerning their size, composition, and surface proteins, presenting a formidable obstacle for SERS-based detection. Addressing this challenge necessitates the development of versatile SERS substrates and analytical methodologies capable of accommodating this diversity. Furthermore, while SERS is renowned for its exceptional sensitivity, consistently achieving low detection limits for exosomes remains a daunting task. The inherent low refractive index and weak Raman scattering signal of exosomes render their detection at low concentrations challenging. Consequently, there is a pressing need to explore innovative SERS substrates that offer heightened sensitivity and to devise advanced signal amplification strategies that can push the boundaries of exosome detection. Thus, our review delves into the significant advancements made in SERS substrates, recognizing their pivotal role in enhancing SERS analysis. Overcoming challenges related to reproducibility, signal uniformity, stability, and biocompatibility has been a primary focus, leading to the development of various innovative methods. For instance, extensive research has been conducted on the shape, size, and core@shell structure of NPs to improve SERS signal consistency and efficiency. Additionally, researchers have explored diverse SERS substrate materials, including porous materials, bacterial substrates, Pt-black SERS templates, and ITO glass, each offering unique properties to enhance performance. Moreover, to further boost SERS signal quality, molecular linkers such as ATPES and antibodies have been developed and employed. These molecular linkers play a critical role in facilitating efficient SERS signal transduction and enhancing the sensitivity and specificity of the analysis.

Last but certainly not least, an essential challenge in SERS-based exosome analysis lies in data analysis. To address this concern effectively, we present a comprehensive overview of data analysis methods, with a particular focus on fingerprint analysis techniques. These methods encompass both linear and non-linear machine learning approaches commonly employed in label-free SERS analysis. Linear machine learning methods, such as PCA, offer valuable insights into the SERS data, allowing for a deeper understanding of underlying patterns and structures within the dataset. On the other hand, deep learning presents tremendous potential, providing enhanced accuracy, automation, and scalability for processing vast and diverse datasets. However, the widespread adoption of deep learning faces challenges, notably the requirement for substantial computational resources and extensive training data. Despite these obstacles, continuous advancements in deep learning techniques and the growing availability of comprehensive SERS-based exosome databases are expected to drive significant progress in this field.

For SERS-based label-free exosome detection to evolve into a practical clinical tool, it must surmount various translational hurdles. These encompass the standardization of protocols, validation across substantial patient cohorts, and seamless integration with established diagnostic workflows. Close collaboration between researchers and clinicians is imperative to bridge the divide between laboratory-based research and clinical implementation. Moreover, SERS instrumentation often comes with a high cost and complexity, which can restrict its accessibility in resource-constrained settings. To confront this challenge, proactive measures should be taken to develop cost-effective SERS platforms and user-friendly devices that can be readily deployed in a diverse array of healthcare settings.

## Figures and Tables

**Table 1:** Comparison between the common exosome isolation methods

Technique	Purity	Equipment	Time	Yield	Specimen volume	Exosome Integrity	Ref
UC	Low (5-25%)	Heavy, not portable	>4 h	Low	High (~1 mL)	Exosome damage	39
SEC	High	Special column	0.3 h	Median	Median (500 μL)	Loss of small vesicle	55
Ultrafiltration	High	Cheap	<4 h	Median	Median (400 μL)	Loss of small vesicle	64
Immunoprecipitation	High (99%)	Expensive	4-20 h	Low	Median (up to 1 mL)	Exosome damage	65
Microfluidic SERS	High	Easy integration	1 h	Median	Low (20 μL)	Exosome damage	50, 66

UC: Ultracentrifugation; SEC: Size Exclusion Chromatography; Microfluidic SERS: Microfluidic Surface-Enhanced Raman Spectroscopy.

**Table 2:** Label-free SERS-based exosome analysis for cancer diagnosis

Cancer type	Exosome resource	Purification	NP size (nm)	EF	EF material	Data analysis	Successful rate	SERS substrate	SERS characteristics	Ref
Breast	Cell	UC	N/A	N/A	N/A	CNN	>95%	AuNPs on silicon wafer	Simple substrate: easy to make, relatively low level of sensitive, uniformity and reproductivity	37
Oral and Oropharyngeal	Salivary	UC	N/A	N/A	N/A	PCA-LDA	74.2%	AuNPs on CaF_2_ glass		39
Multiple	Cell	Commercial kits	N/A	N/A	N/A	Random forest analysis	>96.8%	AuNPs		58
Urogenital	Serum	UC	N/A	N/A	N/A	CNN	>74%	AgNPs on silicon wafer		41
Breast	Cell, serum	SEC/ UC	71.0±9.6	N/A	N/A	N/A	100%/ 79.4%	Au nanostar on a quartz/silicon slide	NPs with different shape: may improve the sensitivity.	36
lung	Bronchoalveolar fluid	SEC	200	N/A	N/A	SVM	N/A	Au nanopyramid		31
Melanoma	Cell	UC	N/A	4.1x10^5^	EV	PLS-DA	N/A	Au@Ag solution	NPs with core-shell structure: may improve the sensitivity. The magnetic SERS platform integrates exosome isolation and Raman signal enhancement	38
Nasopharyngeal	Cell	UC	48± 5	4.57 *10^7^	R6G	Neural network	92.4%	Au@Ag-Si substrate		43
Breast	Cell	Magnetic SERS	50	225	Exosome	PCA	N/A	Magnetic MNP@Au with antibody		50
Breast	Serum from mice	Magnetic SERS	20	N/A	N/A	PCA	N/A	Magnetic Fe_3_O_4_/AuNPs		23
Lung	Fetal bovine serum	UC /commercial kits	N/A	N/A	N/A	PCA	N/A	A hybrid SERS substrate (a graphene covered by Au pyramids)	Tailored substrate materials: relatively complex fabrication, may enhance both the uniformity and sensitivity of SERS substrates.	54
Multiple	Cell, blood	UC	60	3*10^4^	MB	Univariate analysis	93%	Macroporous 3D Au-coated TiO_2_ MIO structures		28
Breast	Cell	UC	22-63	10^4^ to 10^5^	R6G	PCA	N/A	AgNPs grown in bacterial nanocellulose		34
Breast	Cell	UC	100	7.1	Crystal violet	Random forest analysis	89.5%	Pt-black SERS template		35
Lung	Cell	UC	N/A	8.68 *10^6^	R6G	PCA	N/A	3DOM Ag_7_O_8_NO_3_ micropyramids		57
Myeloma	Blood	Commercial kits	< 30	N/A	N/A	PCA	N/A	Microstructured arrays (Si) containing AuNPs		52
Multiple	Plasma	SEC	N/A	4.28 * 10^5^	R6G	TOO	75.9%	AuNPs on APTES glass	Substrates with linking molecules: may enhance the uniformity of the SERS signal.	45
Lung	Cell	SEC	N/A	N/A	N/A	PCA	>90%	AuNPs on APTES glass		32
Lung	Plasma, cell	SEC/ UC	N/A	N/A	N/A	CNN	>90% (cellular) >60% (plasma)	AuNPs on APTES glass		46
Head and neck	Blood, cell	UC/SEC	40-60	N/A	N/A	PCA-LDA/QCA	>97.8%	AuNPs with cysteamine on the quartz microfiber matrix		40
Hepatocellular carcinoma	Blood	UC	N/A	106	Exosome	SVM	>97.56 %	AgNPs with biorecepter		42
Lung	Blood, cell	SEC kit	N/A	N/A	N/A	CNN	93%	AuNPs with antibody		33
Glioma	Cell	UC/filter	N/A	3.4 * 10^5^ (Ag)	EV	Univariate analysis	N/A	Nanobowtiefluidic device (Ag, Au, and Al)	Microfluidic device: may achieve high-throughput separation and enrichment of exosomes.	55

NP: Nanoparticle; EF: Enhancement Factor; CNN: Convolutional Neural Network; PCA: Principal Component Analysis; LDA: Linear Discriminant Analysis; SVM: Support Vector Machine; SEC: Size Exclusion Chromatography; UC: Ultracentrifugation; SERS: Surface-Enhanced Raman Spectroscopy; AuNPs: Gold Nanoparticles; AgNPs: Silver Nanoparticles; EV: Extracellular Vesicles; PLS-DA: Partial Least Squares Discriminant Analysis; R6G: Rhodamine 6G;

**Table 3:** An overview or report of the identified peaks or characteristic signals obtained from Raman spectroscopy analysis of exosomes

Biomolecular	Raman peak (cm^-1^)
Lipid	1049 (C-N, C-C stretching of lipids) 39, 1449 (CH_2_ bending mode of lipids) 39, 1066 (Chain C-C stretching in lipids) 58, 1305 (CH_3_ CH_2_ twisting) 58,1447 (C-H vibration) 58,1450 (CH_2_, CH_3_ def) 58, 970 54, 1070 ((C-C) stretching) 55, 1395 (CH_3_ scissoring) 55, 1435 (CH_2_/CH_3_ scissoring) 55,1455 55,1307 ((C-N) stretching) 38, 1381 (CH3 symmetric) 38, 1465 38
Fatty acids	1286 37, 1140 54
Cholesterol	867 37, 864 37, 1438 54, 2890 ((CH_2_)-Chol stretching) 55, 546 38, 406 38
Phospholipid	826 (C-O-O vibration) 37, 716 (CN-(CH_3_)_3_) 54, 1179 38, 1211 38
Protein	826 37, 1145 37, 1049 (C-N, C-C stretching of Protein) 39, 1149 (CH_2_ bending mode of protein) 39, 1450 (CH_2_, CH_3_ def) 58, 1595 (C = O stretch) 58, 970 54, 1111 54, 1592 54, 1220 (Amide III) 55, 1435 (CH_2_/CH_3_ scissoring) 55, 1620 (ν(C=C) 55, 521 38, 1618 38, 1542 38, 1618 (ν(C=C) 38, 1632 38
Tyrosine	654 37, 1571 37, 638 39, 835 (Asymmetric O-P-O stretching) 58, 1614 54, 660 ((C-C) twisting) 55, 830 55, 1220 55
Proline	939 37,867 37,930 39, 947 58, 1044 (ν_3_PO_4_ 3-(symmetric stretching vibration)) 54
Phenylalanine	630 37, 999 37, 1580 37, 1002 (C-C symmetric stretch of Phenylalanine) 39, 1012 54, 1111 54
Tryptophan	867 37, 1002 (symmetric ring breathing mode) 39, 753 54, 1566 54
Nucleic acid	1100 39, 1323 (CH_2_-CH_2_ of Nucleic Acids) 39, 1577 (guanine) 39, 1655^39^, 816 58,1662 58, 970 54, 1254 55, 1340 (guanine) 55, 786 38, 1243 38
Adenine	631^37^, 736 37, 1571 37, 722 (ν(CC), ν(CO)) 58, 1578 58, 1183 58
Guanine	654 37, 1380 37, 1576 37, 1577 2, 1180 58, 1578 58, 1183 54, 1340 55, 1360 55
Cytosine	1180 58, 1183 54, 1287 54
DNA	722 58, 1510 (ring-breathing modes in the DNA bases) 54, 1592 54 668 38, 1490 38, 1664 38
Saccharide	460 39, 593 39, 835 58, 947 58, 1370 58
Thiocyanate	443 39, 533 39
Phosphate group	1087 28

**Table 4:** Summary of advantages and disadvantages of various data analysis methods

Technique	Principle	Advantage	Disadvantage	Example
Univariate analysis	Interpretation of individual spectral peak independently	Easy interpretation	Without considering the interactions or correlations between different peaks	Peak: Phosphoproteins peak (1087 cm^-1^) 28. Lipid membrane in the CH region (R=*I*_2880_/*I*_2930_) 55
Linear classification method	Unsupervised method, most of them are based on the projection on new variables	Relatively easy to implement, visualize and interpret	Cannot predict new sample	PCA 23, 28, 32, 54, TSNE 63
Linear Discrimination method	Supervised linear method	Can predict new sample	Relatively low accuracy	LDA 39, 40, 42, 49, PLS-DA 38, 49, SVM 31, 42
Deep learning	Artificial neural networks with multiple layers to learn and represent complex patterns in data	Relatively complicated to implement, visualize and interpret	Relatively high level of accuracy	CNN 33, 35-37, 41, 43-48, ANN 36, 48

PCA: Principal Component Analysis; TSNE: t-Distributed Stochastic Neighbor Embedding; LDA: Linear Discriminant Analysis; PLS-DA: Partial Least Squares Discriminant Analysis; SVM: Support Vector Machine; CNN: Convolutional Neural Network; ANN: Artificial Neural Network.

## Abbreviations

EVs: Extracellular Vesicles
SERS: Surface-Enhanced Raman Spectroscopy
EF: Enhancement Factor
Au: Gold
Ag: Silver
NPs: Nanoparticles
UC: Ultracentrifugation
SEC: Size Exclusion Chromatography
TEM: Transmission Electron Microscopy
NTA: Nanoparticle Tracking Analysis
FDTD: Finite-Difference Time-Domain
LSPRs: Localized Surface Plasmon Resonances
PCA: Principal Component Analysis
LDA: Linear Discriminant Analysis
PLS-DA: Partial Least Squares Discriminant Analysis
CNN: Convolutional Neural Network
SVM: Support Vector Machine
3DOM: 3D Ordered Macroporous
MIO: Macroporous Inverse Opal
APTES: 3-Aminopropyl Triethoxy Silane
EF: Epidermal Growth Factor
BP: Backpropagation
ITO: Indium Tin Oxide
DMAP: 4-Dimethylaminopyridine
MB: Methylene Blue
R6G: Rhodamine 6G
4-MBA: 4-Mercaptobenzoic Acid
